# Identification and Functional Analysis of MicroRNAs in Mice following Focal Cerebral Ischemia Injury

**DOI:** 10.3390/ijms161024302

**Published:** 2015-10-14

**Authors:** Cuiying Liu, Lei Zhao, Song Han, Junfa Li, Dongguo Li

**Affiliations:** 1 Institute of Biomedical Engineering, Capital Medical University, Beijing 100069, China; E-Mail: liucy@mail.ccmu.edu.cn; 2 Department of Neurobiology and Center of Stroke, Beijing Institute for Brain Disorders, Capital Medical University, Beijing 100069, China; E-Mail: songhan@ccmu.edu.cn; 3 China-America Institute of Neuroscience, Beijing Luhe Hospital, Capital Medical University, Beijing 101100, China; 4 Department of Anesthesiology Xuan Wu Hospital, Capital Medical University, Beijing 100053, China; E-Mail: zhaoalei@sina.com

**Keywords:** middle cerebral artery occlusion, miRNA microarray, gene ontology, Kyoto encyclopedia of genes and genomes, network

## Abstract

Numerous studies have demonstrated that genes, RNAs, and proteins are involved in the occurrence and development of stroke. In addition, previous studies concluded that microRNAs (miRNAs or miRs) are closely related to the pathological process of ischemic and hypoxic disease. Therefore, the aims of this study were to quantify the altered expression levels of miRNAs in the infarct region 6 h after middle cerebral artery occlusion (MCAO)-induced focal cerebral ischemia in mice using a large-scale miRNAs microarray. Firstly, MCAO-induced cerebral ischemic injuries were investigated by observing the changes of neurological deficits, infarct volume and edema ratio. One hundred and eighteen differentially expressed miRNAs were identified in the infarct region of mice following the MCAOs compared with sham group (*p* < 0.05 was considered as significant). Among these 118 significantly expressed microRNAs, we found that 12 miRNAs were up-regulated with fold changes lager than two, and 18 miRNAs were down-regulated with fold changes less than 0.5 in the infarct region of mice following the 6 h MCAOs, compared with the sham group. Then, these 30 miRNAs with expression in fold change larger than two or less than 0.5 was predicted, and the functions of the target genes of 30 miRNAs were analyzed using a bioinformatics method. Finally, the miRNA-gene network was established and the functional miRNA-mRNA pairs were identified, which provided insight into the roles of the specific miRNAs that regulated specified genes in the ischemic injuries. The miRNAs identified in this study may represent effective therapeutic targets for stroke, and further study of the role of these targets may increase our understanding of the mechanisms underlying ischemic injuries.

## 1. Introduction

Stroke is a leading cause of serious long-term disability in the United States [1]. At present, thrombolytic therapy within a narrow time window is the only acute therapeutic intervention for ischemic stroke, and many clinical stroke trials have failed [2]. Although the effectiveness of neuroprotectants has been demonstrated in rodent experimental stroke models, it has shown a deficiency against stroke in clinical trials because focusing on neuroprotection is not sufficient [3]. As a result, the development of new and effective therapies is urgently required.

MicroRNAs (miRNAs) are endogenous, short, small, non-coding RNA molecules of 21 to 23 nucleotides in length. At present, miRNAs have emerged as key players in physiology, as well as being pathophysiology attributable to its ability to regulate gene expression by either degradation, or translational repression at the post-transcriptional level [4]. Because miRNAs are endogenous and small RNAs, they have attracted much attention recently due to the fact that they have been found to regulate at least 30% of the genes in a cell [5]. Until now, miRNAs have been reported to regulate cellular activities, such as differentiation and development, metabolism, proliferation, and tumorgenesis [6]. Recently, many studies have paid attention to the roles of microRNAs in stroke [7,8,9,10].

In this study, a large scale of miRNA microarray was used to investigate the miRNA expression profiles in the infarct region of mice following middle cerebral artery occlusion (MCAO)-induced focal cerebral ischemia. Then, a bioinformatics analysis using Gene Ontology (GO) and the Kyoto Encyclopedia of Genes and Genomes (KEGG) databases were applied to interpret the function of the genes targeted by the differentially expressed miRNAs. The aims of the study were to identify the key miRNAs induced by ischemic stroke, which will provide the new biomarker for clinical therapy for stroke.

## 2. Results

### 2.1. Ischemia-Induced Cerebral Injuries of Mice

**Figure 1:**
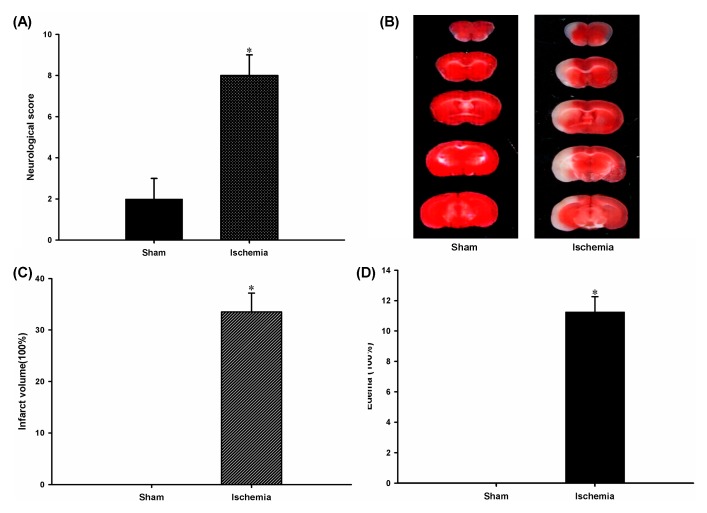
MCAO induced mice cerebral injuries. (**A**) Statistical results of neurological score from the Sham and Ischemia groups (*n* = 6 per group). * *p* < 0.05; (**B**) Representative photographs of TTC-stained coronal brain sections of mice from the Sham and Ischemia group; (**C**) Statistical results of infarct volume from the sham and Ischemia groups (*n* = 6 per group). * *p* < 0.05; and (**D**) Statistical results of edema ratio from the Sham and Ischemia groups (*n* = 6 per group). * *p* < 0.05.

The neurological score of the MCAO group was obviously higher than that in the sham group (Figure 1A). The 2,3,5-triphenyltetrazoliumchloride (TTC) staining result indicated that a large area of infarction was observed in the cerebral cortex and striatum of the Ischemia group (Figure 1B). Similarly, the statistical results of the infarct volume and edema ratio remarkably increased after 6 h of MCAO when compared with those of the sham group (Figure 1C,D), respectively.

### 2.2. Differential Expression of MiRNAs in the Brains of the Mice following Middle Cerebral Artery Occlusion

**Figure 2:**
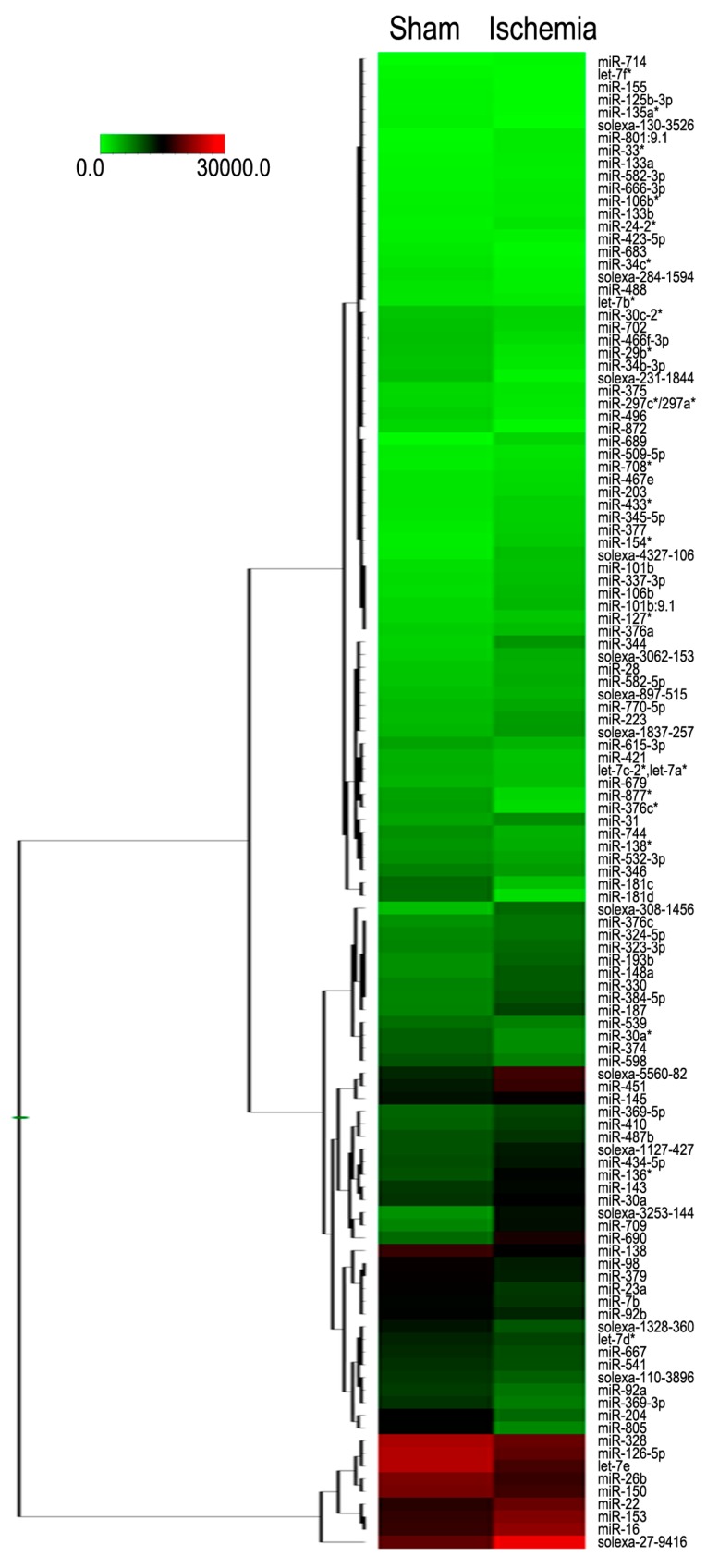
Hierarchical cluster analysis of the altered miRNAs in the infarct region of the MCAO mice. An asterisk following the name indicates a miRNA expressed at low levels relative to the miRNA in the opposite arm of the precursor. The color code in each heat map is linear, with green as the lowest, and red as the highest. The average signals of the changed miRNAs in each of the two groups were clustered using a Euclidean distance function. The miRNAs with the most similar expression patterns were placed next to each other (*n* = 12 per group).

**Figure 3:**
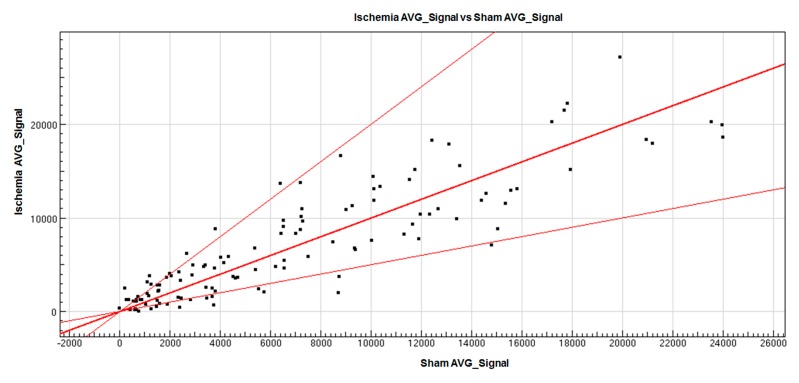
The scatter plots of gene expression pattern in the Ischemia group. Axis *X* represents the average signals of the sham group, and Axis *Y* is the average signals of the Ischemia group. Each spot represents the average signal of one gene. Three straight lines represent that the average signal ratios of the spots on the line in the two types of tissues were 2, 1, and 0.5, respectively, from the top to down. The spots above the line of ratio 2 are the significantly up-regulated genes, and spots below the line of ratio 0.5 are significantly down-regulated genes. The farther the spot is from the line, the more significant difference in miRNA expression is.

To characterize the miRNA expression profile in cerebral injuries, a miRNA microarray assay was performed. According to the results from the microarray analysis, a total of 118 miRNAs changed dramatically in the infarct core region of the MCAO mice (*n* = 12, *p* < 0.05 compared with sham group). Of the total, 61 miRNAs were up-regulated, while 57 miRNAs were down-regulated in the infarct core region of the MCAO mice when compared with that of the sham group (*n* = 12). The hierarchical clustering analysis of the changed miRNAs was shown in Figure 2. The expression level of these 118 microRNAs in Sham and Ischemia groups was shown in Table S1. Furthermore, it can be seen that in the scatter graph of the miRNA distribution, which is based on their fold changes in expression (Figure 3), the majority of the altered miRNA fell out the range of the two fold up or down regulation. Of note, the expression of 30 miRNAs altered more significantly with 12 up-regulations for fold changes lager than two and with 18 down-regulations for fold changes less than 0.5 in the cerebral cortexes of the MCAO mice, when compared with that of the sham group. We then further focus on the 30 significantly expressed miRNAs in the next step.

### 2.3. Verification of MiRNA Expression via qRT-PCR

**Figure 4:**
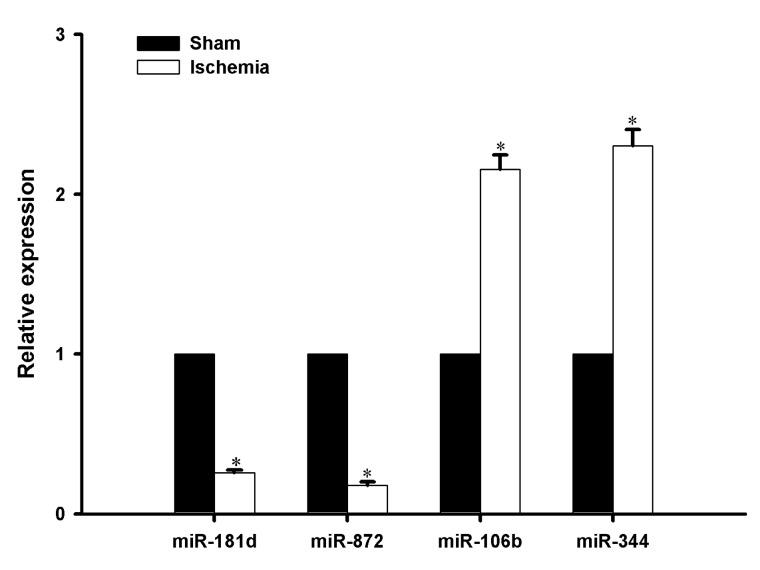
Gene expressions of four specified miRNAs are confirmed by real time RT-PCR. Each corresponding control of the verified miRNA with change is normalized as 1. Each test was in triplicates and U6 was used as internal control. The bars represent the mean ± SE, and *n* = 6 per group. * *p* < 0.05.

To further confirm the accuracy of the miRNA microarray results, the miR-181d, miR-872, miR-106b, and miR-344 of 30 miRNAs were verified by qRT-PCR. As shown in Figure 4, miR-181d and miR-872 were down-regulated with a 0.5-fold change in the infarct region of the MCAO mice, while miR-106b and miR-344 were up-regulated with two fold changes in the infarct region of the MCAO mice. The expression of these microRNAs was consistent with the expression patterns in microarray results.

### 2.4. Gene Ontology and Kyoto Encyclopedia of Genes and Genomes Pathway Analyses of the Target Genes of 30 Specified MiRNAs

**Figure 5:**
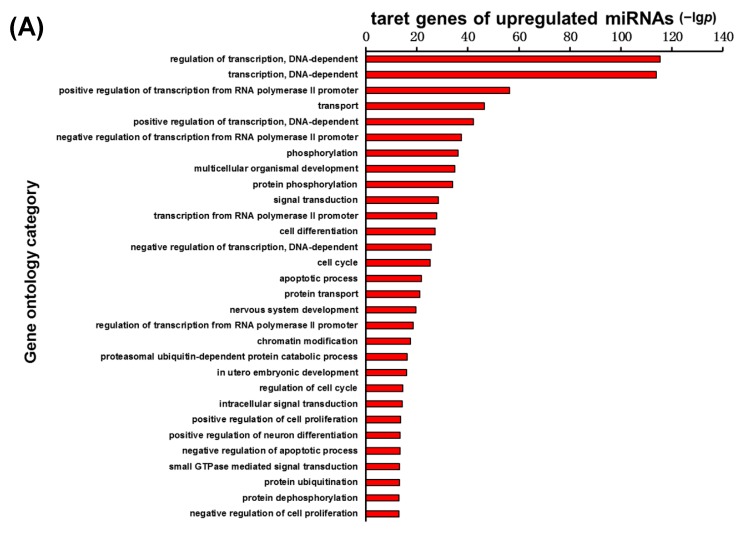

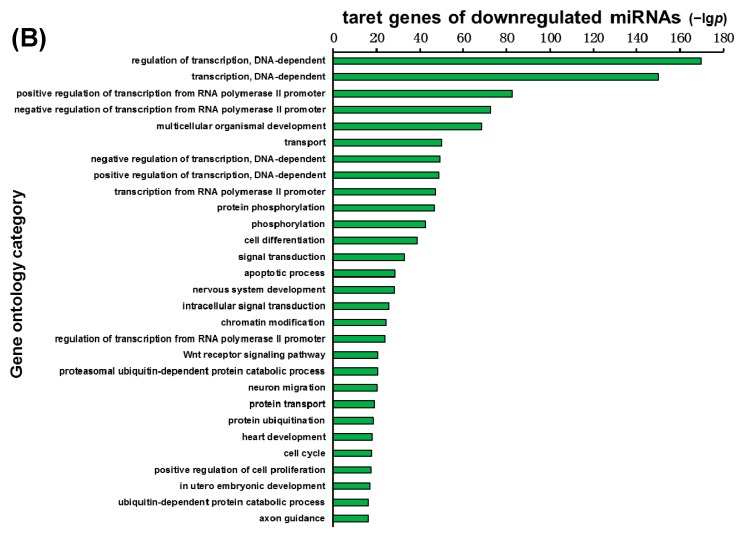
Target genes of the up-regulated miRNAs (**A**) and down-regulated miRNAs (**B**) annotated significant GOs. The vertical and horizontal axes are the GO terms and the −lg*p* of GOs, respectively. lg*p* is the logarithm of the *p* value and *p* < 0.05 is considered significant.

**Figure 6:**
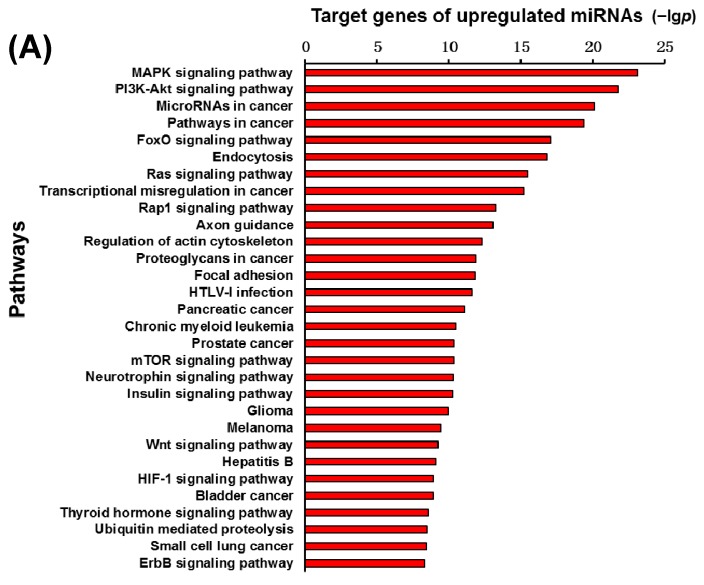

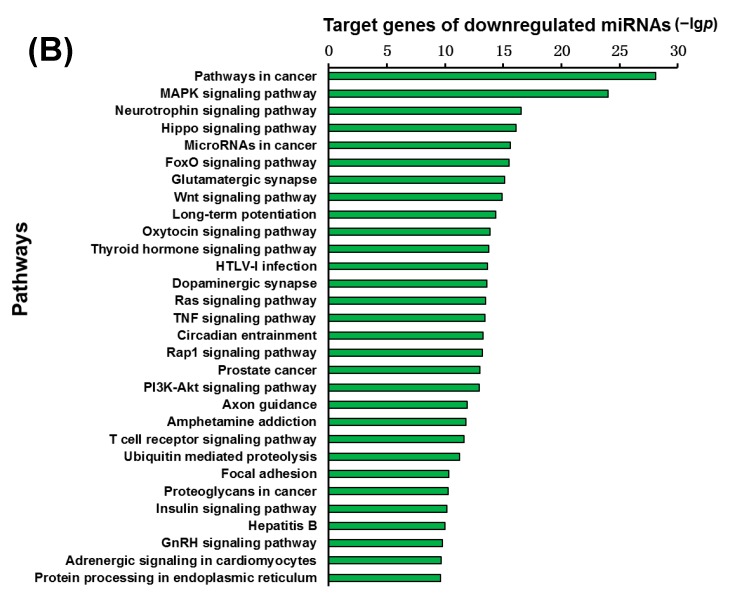
Target genes of the up-regulated miRNAs (**A**) and down-regulated miRNAs (**B**) annotated significant pathways. The vertical and horizontal axes are the pathway terms and the −lg*p* of pathways, respectively. lg*p* is the logarithm of *p* value and *p* < 0.05 is considered significant.

To clarify the function of 30 specified miRNAs in the infarct core region of the MCAO mice, the genes targeted by these miRNAs were predicted using a TargetScan and miRanda, respectively, and then the concurrent target genes were selected from these two methods for further analysis. In this study, the significant molecular function was first interpreted for these target genes regulated by the 30 specified miRNAs using GO analysis. Figure 5A showed the most significant 30 GO categories of the target genes regulated by the up-regulated microRNAs, while Figure 5B illustrated the most significant 30 GO categories of the target genes regulated by the down-regulated microRNAs. The function of the genes regulated by the up-regulated microRNAs was related to the regulation of transcription, DNA-dependent, phosphorylation, cell cycle, apoptotic process, and so on. The function of the genes regulated by the down-regulated microRNAs was related to the function of the regulation of transcription, DNA-dependent, protein phosphorylation, and apoptotic process. Then, another functional analysis was applied to the 30 specified miRNAs target genes involved in the significant pathways via a KEGG database. Figure 6A,B illustrated the significant pathways annotated by the target genes of the up-regulated microRNAs and down-regulated microRNAs, respectively. The MAPK signaling pathway and PI3K-Akt signaling pathway are ranked at top of the Figure 6A. The pathways in cancer, MAPK signaling pathway, and Neurotrophin signaling pathway, are at the top of Figure 6B.

### 2.5. Construction of Functional MiRNA–Gene Network

**Figure 7:**
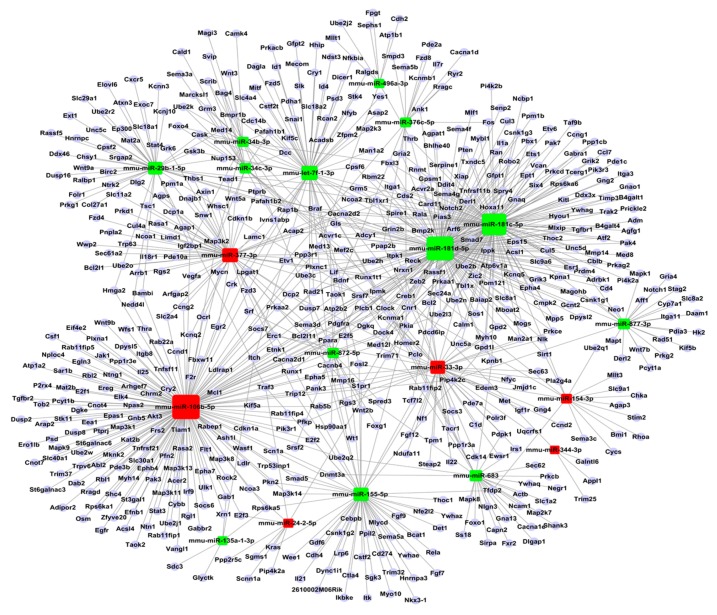
The miRNA-gene-network of the specified miRNAs. The squares indicate the miRNA, while red indicates the up-regulated miRNAs, and green indicates the down-regulated miRNAs.

Additionally, the network of miRNAs-target proteins was constructed according to the regulated relationship between the miRNAs and the target genes. As shown in Figure 7, it can be directly seen that specified miRNAs may be involved in ischemic injury by way of targeting the corresponding genes. Table 1 shows the miRNAs regulated genes with degrees above 50, which indicates that these miRNAs, such as mmu-miR-106b-5p, may play important roles in ischemic injury. Furthermore, the genes regulated by miRNAs with degrees above 5 are shown in Table 2. For example, creb1 can be regulated by six microRNAs.

**Table 1:** The miRNAs regulated genes with degrees above 50.

MicroRNA	Style	Degree
mmu-miR-106b-5p	up	166
mmu-miR-181d-5p	down	158
mmu-miR-181c-5p	down	136
mmu-let-7f-1-3p	up	61
mmu-miR-377-3p	down	60
mmu-miR-155-5p	down	52
mmu-miR-33-3p	up	51

**Table 2:** The genes regulated by miRNAs with degrees above 5.

Gene Symbol	Description	Degree
Creb1	cAMP responsive element binding protein 1	6
Rap1b	RAS related protein 1b	6
Acvr1c	activin A receptor, type IC	5
Tbl1xr1	transducin (β)-like 1X-linked receptor 1	5

## 3. Discussion

At present, microRNAs have emerged as vital regulators in many physiological and pathological processes, including ischemic and hypoxic damage. In regards to cerebral ischemic injuries, one group presented the profiling of miRNA in the blood and brain during I/R in a rat MCAO model, and identified distinct regulation patterns for seven clusters of miRNA, which controlled the expression of four genes known to be important in the progression of cerebral ischemia [11]. Dharap *et al.* profiled miRNAs in the brains of adult rats as a function of reperfusion time after transient MCAO, and found that those mRNAs regulated by altered miRNAs mediated inflammation, transcription, neuroprotection, receptor function, and ionic homeostasis using bioinformatics analysis [12]. However, these reports did not distinguish the ischemic core and peri-infarct region. In this study, the different regions in the brains of the MCAO mice were distinguished. Therefore, the differences in miRNAs changes between this study and previous studies may be derived from the differences in the species, selected brain regions, and concrete MCAO experimental procedures. Currently, many miRNAs have been considered as innovative targets for cerebral ischemia and stroke [13,14].

In this study, 12 miRNAs were up-regulated, and 18 miRNAs were down-regulated, with 2- and 0.5-fold changes in the infarct region of the mice following 6 h of MCAO (*n* = 12 per group), and were compared with the sham group. The expression of some of the specified miRNAs identified in this study has been reported to be involved in different biological processes. For example, it was observed that miR-106b was significantly up-regulated in the ischemic injured brains; MiR-106b was found to be associated with a high risk of recurrence of breast cancer; and miR-106b was a putative plasma marker for the risk assessment of patients with breast cancer [15]. Similarly, another study found that the miR-106b expression was elevated in higher stage tumors, and was correlated with tumor progression in breast cancer patients [16]. Another study has demonstrated that miR-106b promoted the proliferation and invasion of laryngeal carcinoma cells by directly targeting RUNX3 [17]. Moreover, miR-181c modulates the proliferation, migration, and invasion of neuroblastoma cells by targeting Smad7, while miR-181d acts as a tumor suppressor in glioma by targeting K-ras and Bcl-2 [18,19]. The available data have shown that miR-155 plays an important role in the proper homeostasis of immune regulation, as well as the suppression of oncogenesis [20]. Additionally, one study found that miR-344 inhibited the adipocyte differentiation via targeting GSK3β, and subsequently activating the Wnt/β-catenin signaling pathway [21]. Another study found that miR-344 was commonly down-regulated in Huntington’s disease models [22]. It has been reported that the increased expression of miR-714 in VSMCs may be involved in VSMC calcification by disrupting Ca^2+^ efflux proteins [23]. The concordant elevation of miR-714 was also found in the plasma and kidneys at 3, 6, and 24 h following acute kidney injuries, when compared to the sham-operated mice [24]. Wang determined that miR-377 functions as a tumor suppressor in human clear cell renal cell carcinoma by targeting ETS1 [25]. At the present time, miR-496, miR-805, and miR-872 have not been extensively investigated. However, they may be related to fetal alcohol syndrome, renal ischemia reperfusion injury, and diabetic cardiomyopathy in Akita, respectively [26,27,28]. Currently, there are also few reports available regarding the miRNAs labeled with asterisks, such as miR–miR-135a* and miR–miR-29b*, and their functions are unclear possibly due to their low expression.

In order to interpret the functions of miRNAs, it was crucial to predict the specified miRNAs target genes by using bioinformatics technology. In this study, the specified miRNAs target genes were predicted via TargetScan and miRanda. Due to the fact that a single miRNA can target hundreds of genes, it is most likely that multiple significant biological processes, molecular functions, and pathways are involved in the ischemic injuries. Therefore, the biological processes of these genes targeted by 30 specified miRNAs were first analyzed using a GO database. The results indicated that the function of the genes regulated by the up-regulated miRNAs was related to the regulation of transcription, DNA-dependent, phosphorylation, cell cycle, apoptotic process, and so on. The genes regulated by the down-regulated miRNAs were related to the function of the regulation of transcription, DNA-dependent, protein phosphorylation and apoptotic process. Next, a KEGG database was applied to analyze the significant pathways of these genes targeted by 30 specified miRNAs. From the KEGG analysis, the significant pathways of the target genes of the up-regulated miRNAs and down-regulated miRNAs were annotated, respectively. The MAPK signaling pathway and PI3K-Akt signaling pathway were ranked at the top in the Figure 6A. The pathways in cancer, and the MAPK and Neurotrophin signaling pathways were at the top of Figure 6B.

Moreover, the miRNA-gene network was constructed according to the relationship between the miRNAs and the targeted genes, which indicated the direct interaction between the miRNA and the gene. Among them, the genes regulated by the miRNAs with degrees above 5 are shown in Table 2. Creb1, which belongs to the CREB/ATF family of transcription factors, was predominantly located in the nucleus bound to CRE, and was activated by phosphorylation at Ser133 in the kinase-inducible domain [29]. The Ser-133 phosphorylation sites of the cAMP-response element binding protein (CREB) was a key gene which mediated a variety of downstream transcription initiation factors, regulated neuronal survival, and promoted the expression of a large number of genes [30]. As a small GTPase, the active GTP-bound form of Rap1B(Rap1b) was able to bind to a large number of effector proteins, and in doing so transmit signals to the downstream components of the signaling pathways [31]. The previous study found that the specific activation of Rap1B contributed to the neuronal polarization via interactions with RalA and Nore1A in addition to PI3-kinase [32]. Moreover, one group demonstrated that TBL1XR1 induced lymphangiogenesis and lymphatic metastasis in esophageal squamous cell carcinoma (ESCC) via the up-regulation of VEGF-C, and may represent a novel prognostic biomarker and therapeutic target for patients with ESCC [33]. Furthmore, previous studies have found that cholinergic biomarkers show relevance for ischemic stroke and a group of miRNAs target cholinergic genes [34,35]. Therefore, to test whether the miRNAs identified by us could target cholinergic genes, may support a thread for stroke treatment. Because each of the target prediction algorithms had false-negative or false-positive predictions due to the complexity of the miRNA-target interactions, it was important to further verify the regulatory relationship between the miRNA and its target genes.

## 4. Experimental Section

### 4.1. MCAO-Induced Focal Cerebral Ischemia Mouse Model

Adult (8 to 10 weeks of age) male BABL/c (Bagg albino inbred “c” strain) mice (weighing 18 to 22 g), were purchased from the Experimental Animal Center of the Chinese Academy of Medical Sciences, PR China. These mice were housed at room temperature (18 to 22 °C) with 12 h light/dark cycles, where they received food and water *ad libitum*. All of the procedures in this study were conducted according to the guidelines set by the University Animal Care and Use Committee of the Capital Medical University (Permit Number: 2011-X-026), and they were also consistent with the NIH Guide for the care and use of laboratory animals (NIH Publications No. 80–23).

The MCAO-induced permanent focal cerebral ischemia mouse model was prepared as previously reported [10,36]. The left common artery and left external carotid artery were exposed, and then ligated through a ventral midline neck incision under anesthesia with pentobarbital sodium (0.06 g/kg i.p.). Then, a 4–0 surgical nylon filament with a blunt tip (0.23 mm in diameter) was inserted into the internal carotid artery to a point approximately 12 mm distal to the carotid bifurcation to occlude the origins of the middle cerebral artery. Flow doppler measurement was used to monitor the cerebral blood flow after MCAO. In the sham group, the mice received the same surgical exposure of the carotid arteries without occlusion. During the surgery, the body temperatures were maintained by using a heating lamp and thermal blanket (rectal temperature was maintained at 38.0 °C). The mice were placed in a post-operative cage, and were kept warm and undisturbed for at least of 2 h for observation purposes. The mortality rate was less than 5%.

The brains of the mice were removed 6 h after the MCAOs, and then were immediately placed into ice-cold artificial cerebral spinal fluid (ACSF, in mM: NaCl 125.0, KCl 2.5, CaCl_2_ 2.0, NaHCO_3_ 26.0, NaH_2_PO_4_ 1.25, MgCl_2_ 1.0, glucose 5.0, pH 7.4) bubbled with 95% O_2_, and 5% CO_2_. The cortexes from the indicated regions were collected according to the previously reported method [37]. To summarize, 2 mm from the anterior tip of the frontal lobes were cut and the left side of brains were sectioned into four 2 mm-slices. Then, longitudinal cuts approximately 1 mm from the midline through each hemisphere were made in order to remove the tissue supplied by anterior cerebral artery. Transverse diagonal cuts were then made at an approximately 45 degree angle position in order to separate the infarct core. The tissue samples were rinsed with diethypyrocarbonate (DEPC) water and instantly placed into RNase-free tubes to be frozen in liquid nitrogen cans.

### 4.2. Neurological Deficit Measurements

The neurological deficits of the mice were scored according to the neurological disability status scale (NDSS) reported by Rodriguez *et al.* [38] 6 h after the MCAOs. The NDSS contained 10 progressive steps beyond 0 (normal), and extended to status 10 (death). Accordingly, the six major steps indicated the following: 0 represented normal (no neurological dysfunction), and 2 represented a slight decrease in mobility, along with the presence of passivity. Category 4 represented moderate neurological dysfunction, and included additional alterations, such as moderate hypomobility, flattened posture, lateralized posture, hunched back, ataxic gait, decreased body tone and muscular strength, and slight motor incoordination. Category 6 corresponded to more handicapped animals; however, these animals were still able to walk, with more marked hypomobility, circling, tremor, jerks and/or convulsions, forelimb flexion, and moderate motor incoordination. Category 8 corresponded to respiratory distress, and total incapacity of movements and coordination. Status 10 referred to death due to MCAO. In all of the cases, where the criteria for the precise grade were not met, the nearest appropriate number was utilized, for example 1, 3, 5, 7, and 9.

### 4.3. Evaluation of Ischemic Infarct and Edema

Immediately following the evaluation of the neurological deficits, double blind measurements of the infarct volume and edema were taken. The mice were sacrificed 6 h after the MCAO, and their brains was quickly removed and cut into 1.5 mm thick coronal sections. The brain sections were incubated for 20 min in a solution of 0.5% 2,3,5-triphenyltetrazolium chloride (TTC) in 0.01 M phosphate buffered saline at 37 °C and then the slices were scanned into a computer. The images were analyzed using Image Pro Plus1 6.0 software (Media Cybernetics, Silver Spring, MD, USA) according to the ischemic area evaluation procedure reported by Wexler *et al.* [39]. The best-fit equalization option from the Image Pro Plus^®^ (Media Cybernetics, Silver Spring, MD, USA) was applied to the image prior to the analysis. The edema was calculated using the equation:

*E* = (ΣVL − ΣVR)/(ΣVL + ΣVR) × 100% (1)

where, ΣVL and ΣVR are the volume of the left and right hemispheres, respectively. The background was calculated using the following equation: B = ΣVS/ΣVT × 100%, where, ΣVS is the volume of the unstained white matter in the sham the group, and ΣVT is the total brain volume. In order to account for the effects of the edema and background, the infarct size was indirectly estimated and expressed as a percentage of the total brain using the equation: I = [ΣVI × (1 − E)/ΣVT × (1 − B)] × 100%, where, ΣVI is the volume of tissue that is not stained with the TTC of the MCAO mice.

### 4.4. MiRNA Microarray

The total RNA, including the miRNAs from the cerebral cortexes of the mice was extracted by using a *mir*Vana™ miRNA Isolation Kit (Ambion Inc., Austin, TX, USA) according to the manufacturer’s instructions. NanoDrop ND-1000 spectrophotometry (NanoDrop Tech, Wilmington, DE, USA) and an agarose gel electrophoresis were used to determine the concentration and integrity of the RNA, respectively.

At this point, a miRNA microarray was performed according to the MicroRNA Expression Profiling Assay Guide (Illumina Inc., San Diego, CA, USA). The assay started by adding a stretch of poly A tail to the 3ʹ end of each sequence in the 1000 ng intact total RNA sample. Following the conversion, extension, amplification, and labelling of the RNAs, the product was hybridized to the BeadChip, which contained 656 probes from the version of mouseMI_V2_R0_XS0000129-MAP (Illumina Inc.) using a hybridization chamber. After the BeadChips were washed, the Illumina BeadArray Reader (Illumina Inc.) was then used to record the images of the BeadChip sections in high-resolution.

The data from the images were analyzed by GenomeStudio™ Gene Expression Module v1.0 software (Illumina Inc.). Compared with the background, the genes’ detection *p* value <0.01 was considered to be accurately detected and was selected for further analysis. The microarray signal was analyzed after subtracting the background, and then normalized by using the normalization algorithms of Quantile.

### 4.5. Real-Time Quantitative Reverse Transcription Polymerase Chain Reaction (qRT-PCR)

Four differentially expressed miRNAs **(**miR-181d, miR-872, miR106b, and miR-344) were validated using the miRCURY LNA™ Universal RT microRNA PCR (Exiqon A/S, DK-2950 Vedbaek, Denmark). A real-time PCR amplification was performed with a Mx3000P™ (Agilent Technologies Inc., Santa Clara, CA, USA) as follows: polymerase activation/denaturation (95 °C for 10 min), 40 amplification cycles (95 °C for 10 s and 60 °C for 1 min).

### 4.6. Gene Ontology (GO) and Kyoto Encyclopedia of Genes and Genomes (KEGG) Analysis

The TargetScan and miRanda were applied to predict the target genes of specified miRNAs obtained in this study. Then, a GO analysis was applied to annotate the specified miRNAs target genes, which participated in the significant biological processes and molecular functions [40]. Specifically, the two-side Fisher’s exact test, and a χ^2^ test were used to classify the GO category, and the threshold of significance was defined by the *p*-value (*p* < 0.05). Furthermore, the false discovery rate (FDR) was also calculated to correct the *p*-value. The smaller the FDR indicates the smaller the error in judging the *p*-value.

Similarly, a pathway analysis was used to find out the significant pathway of the differential genes according to the KEGG, Biocarta, and Reatome. However, this study still turned to the Fisher’s exact test, and the χ^2^ test to select the significant pathways, and the threshold of significance was defined by the *p*-value with FDR to correct.

### 4.7. MicroRNA-Gene-Network

In order to build a miRNA-gene-network, the relationship between the miRNAs and genes was calculated by their differential expression values, and according to their interactions in the Sanger miRNA database. The degree was the contribution of one miRNA to the surrounding genes, or the contribution of one gene to the surrounding miRNAs. The key miRNA and genes in the network consistently had the highest degrees.

### 4.8. Statistical Analysis

The statistical analysis was conducted by a one-way analysis of the variances (ANOVA), followed by all of the pairwise multiple comparison procedures, using a Bonferroni test. All of the data were presented as mean ± SE. Significance was regarded as at least *p* < 0.05.

## 5. Conclusions

This study demonstrated that the MCAO mice were associated with an altered miRNA profile, which will assist in the understanding of the role of the miRNAs in strokes. As miRNAs hold great potential as therapeutic targets, the miRNAs identified in this study may also assist in understanding of the mechanism of MCAO, and in determining a new molecular target for future clinical therapy or biomarkers for strokes.

## Supplementary Materials

Supplementary materials can be found at http://www.mdpi.com/1422-0067/16/10/24302/s1.
